# The predictive value of ERCC1 and p53 for the effect of panobinostat and cisplatin combination treatment in NSCLC

**DOI:** 10.18632/oncotarget.3620

**Published:** 2015-04-29

**Authors:** Yang Cai, Xiang Yan, Guoqing Zhang, Weihong Zhao, Shunchang Jiao

**Affiliations:** ^1^ Department of Medical Oncology, Chinese PLA General Hospital, Beijing 100853, China

**Keywords:** panobinostat, cisplatin, ERCC1, gain of function mutant p53, NSCLC

## Abstract

Cisplatin is one of the most common chemotherapeutic drugs for non-small cell lung cancer (NSCLC). However, the response rate is limited because of drug resistance. Histone deacetylase inhibitors (HDACis), which can alter DNA accessibility by regulating chromatin structure and inducing apoptosis, exhibit a synergistic action with cisplatin. However, no biomarkers that can predict the efficacy of the combination of HDACis and cisplatin have been reported. Our study found that panobinostat, an HDAC inhibitor, increased the cisplatin sensitivity of several NSCLC cell lines with low ERCC1 expression but not those with high ERCC1 expression or gain-of-function (GOF) p53 mutation despite of ERCC1 expression level. ERCC1 knockdown increased the cisplatin sensitivity of NSCLC cell lines with high ERCC1 expression without GOF p53 mutations. In addition, in low ERCC1 expression NSCLC cell lines, knockdown of GOF mutant p53 enhanced cisplatin sensitivity. Further double knockdown of ERCC1 and GOF mutant p53 but not ERCC1 knockdown alone increased the cisplatin sensitivity of cells with both high ERCC1 expression and GOF p53 mutations. Therefore, this study demonstrated that ERCC1 expression combined with p53 mutation status may determine the efficacy of cisplatin and HDACi combined therapy and guide the development of future NSCLC therapies.

Lung cancer is the leading cause of cancer death worldwide and non-small cell lung cancer (NSCLC) accounts for more than 85% of all lung cancer cases [1]. Although significant progress has been made in lung cancer treatment over the last 10 years, platinum-based chemotherapy is still the most common treatment. However, because primary and acquired drug resistance limit chemotherapy's efficacy, only approximately 16% of all lung cancer patients live for 5 years or more after diagnosis [2]. Therefore, new approaches to enhance sensitivity and reverse drug resistance in cancer treatment are urgently needed.

Epigenetic alterations such as DNA methylation, mRNA regulation and other posttranslational changes in chromatin have emerged as novel targets in cancer treatment. Among them, the balance between histone transacetylases and deacetylases, which mediate the expression of tumor suppressor genes and oncogenes, makes these enzymes promising therapeutic targets [3]. Previous studies have shown that histone deacetylases (HDACs) are important for gene expression because HDAC levels vary in different cancer types, and promising results with HDAC inhibitors (HDACis) combined with chemotherapeutic drugs in cancer treatment have been reported [4, 5]. Two HDACis, vorinostat [6] and romidepsin [7], have been approved by the US FDA for the treatment of cutaneous T-cell lymphoma (CTLC), and another HDACi, istodax, has been approved for the treatment of peripheral T-cell lymphoma over the last ten years [7].

The antitumor mechanisms of HDACis primarily include altering DNA accessibility by regulating chromatin structure and inducing apoptosis by up-regulating acetylated p53 and p21 [8, 9]. Panobinostat, an HDACi in the hydroxamate family, has been shown in phase I clinical trials to be effective against leukemia and solid tumors, and it is currently being investigated in phase II and III clinical trials [10–12]. Panobinostat also has been confirmed to have a synergistic effect with cisplatin by many preclinical studies [13–17].

Excision repair cross-complementation group 1 (ERCC1) is a critical protein involved in nucleotide excision repair (NER), and ERCC1 expression reflects DNA repair capacity and clinical drug resistance, especially cisplatin resistance [18]. Wild-type p53 is required for the induction of apoptotic cell death by certain antitumor drugs, such as HDACis and anthracycline [19, 20]. Therefore, in this study, we investigated whether ERCC1 and p53, both of which are important biomarkers related to drug sensitivity, have predictive roles for the efficacy of combined panobinostat and cisplatin treatment. In addition, we also sought to elucidate the mechanisms of ERCC1 and p53 in determining the sensitivity of NSCLC cell lines to panobinostat and cisplatin.

## RESULTS

### ERCC1 expression and p53 status in 8 NSCLC cell lines and their sensitivity to cisplatin

**Figure 1:**
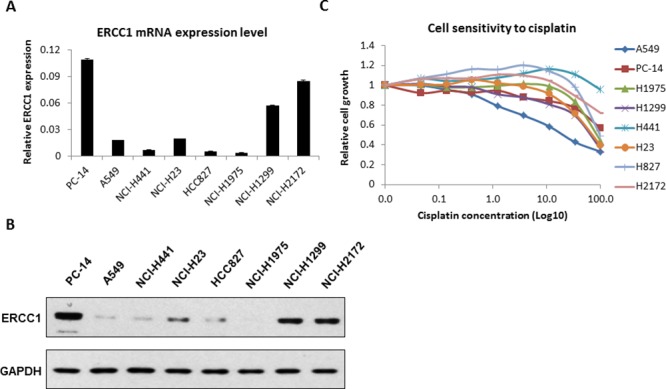
ERCC1 expression and cell sensitivity to cisplatin in 8 NSCLC cell lines **A.** Total RNA was isolated from 8 NSCLC cell lines. ERCC1 expression was analyzed by RT-qPCR. The data are expressed as the mean ± SD. **B.** The protein level of ERCC1 in these cell lines was analyzed by western blot. The relative expression level was quantified by density scan. **C.** The 8 cell lines were treated with cisplatin at different doses for 48 hours. Cell viability was assessed using Cell-Titer Glo. The data are expressed as the mean ± SD. The values are from the average of three independent experiments.

First, we examined the expression levels of ERCC1 in 8 NSCLS cell lines: A549, PC14, NCI-H23, HCI-H441, HCC827, NCI-H1299, NCI-H1975 and NCI-H2172. ERCC1 expression in these cell lines was analyzed by reverse transcription (RT)-qPCR (Fig. 1A) and western blot (Fig. 1B) assays. We found high ERCC1 expression levels in PC14, H1299 and H2172 cells and low levels in the other 5 cell lines. The p53 status of these cell lines was also investigated. Then, we classified these 8 cell lines into different categories according to their ERCC1 level and p53 status. A549 and H827 were classified as ERCC1^Low^/p53^WT^ cell lines. H23 and H441, which harbor non-gain-of-function (GOF) p53 mutations, were classified as ERCC1^Low^/p53^MU^ cell lines. H1299 and H2172, which do not express p53, were classified as ERCC1^High^/p53^Null^ cell lines. Similarly, H1975 was classified as a ERCC1^Low^/p53^GOF^ cell line, and PC14 was classified as a ERCC1^High^/p53^GOF^ cell line. The information on the 8 cell lines is listed in abbreviated form based on ERCC1 expression level and p53 status in Supplementary Table S1.

Next, we examined the cisplatin sensitivity of the NSCLC cells. We treated all 8 cell lines with different doses of cisplatin from 0 to 100 μM for 48 hours, and cell proliferation was measured using Cell Titer-Glo. As shown in Fig. 1C, A549 cells were the most sensitive to cisplatin, with an IC_50_ of approximately 12 μM. However, the other 7 cell lines all exhibited resistance to cisplatin independent of their ERCC1 expression level. The IC_50_ of these 7 lines was higher than 50 μM. There was no obvious relationship between ERCC1 expression and the effect of cisplatin in these cells.

### Effect of panobinostat on the 8 NSCLC cell lines

**Figure 2:**
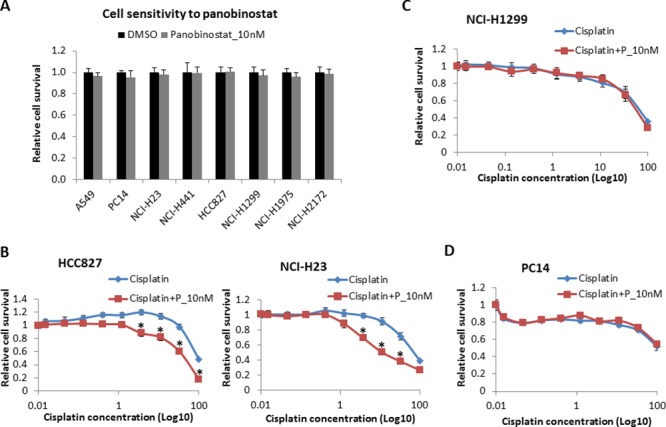
Cell sensitivity to panobinostat and to panobinostat combined with cisplatin **A.** All 8 cell lines were treated with panobinostat at 10 nM for 48 hours. Cell viability was assessed using Cell-Titer Glo. The data are expressed as the mean ± SD*. All 8 NSCLC cell lines were also treated with 10 nM panobinostat combined with different doses of cisplatin for 48 hours. Cell viability was assessed using Cell-Titer Glo. The data are expressed as the mean ± SD *, *p* < 0.05, *t* test. The data plots are as follows: **B.** HCC827 and NCI-H23 cells; **C.** NCI-H1299 cells; and **D.** PC14 cells. P represents panobinostat. All the values are from the average of three independent experiments.

Panobinostat was selected to treat the NSCLC cells because of its high potency. In our previous study, panobinostat itself did not kill tumor cells but sensitized cisplatin below 20 nM after 48 hours of treatment (data not shown). Here, we treated the NSCLC cells with panobinostat at 10 nM. After 48 hours of treatment, cell viability was measured, and 10 nM panobinostat had a less than 5% inhibition rate in all cancer cell lines (Fig. 2A).

### Panobinostat enhances cisplatin sensitivity in ERCC1 low-expressing cells without GOF p53 mutations

In this assay, 10 nM panobinostat was combined with cisplatin at different doses from 0 to 100 μM to treat all 8 cell lines for 48 hours. In H827 (Fig. 2B, left panel) and A549 (Supplementary Fig. S1A) cells, which are ERCC1^Low^/p53^WT^, as well as in H23 (Fig. 2B, right panel) and H441 (Supplementary Fig. S1B) cells, which are ERCC1^Low^/p53^MU^ cell lines, panobinostat significantly improved the cytotoxicity of cisplatin. However, no such effect was observed in the other 4 cell lines: H1299 (Fig. 2C) and H2172 (Supplementary Fig. S1C) cells, which are ERCC1^High^/p53^Null^ cell lines, or in PC14 (Fig. 2D) and H1975 (Supplementary Fig. S1D) cells, which are p53^GOF^ cell lines. Three of the four resistant cell lines exhibited high ERCC1 expression, while all 4 of the sensitive cell lines had low ERCC1 expression levels. The results suggest that the ERCC1 expression level can be considered a predictive marker of resistance to cisplatin combined with panobinostat.

### ERCC1 knockdown or over-expression affects sensitivity to cisplatin combined with panobinostat in NSCLC cell lines

**Figure 3:**
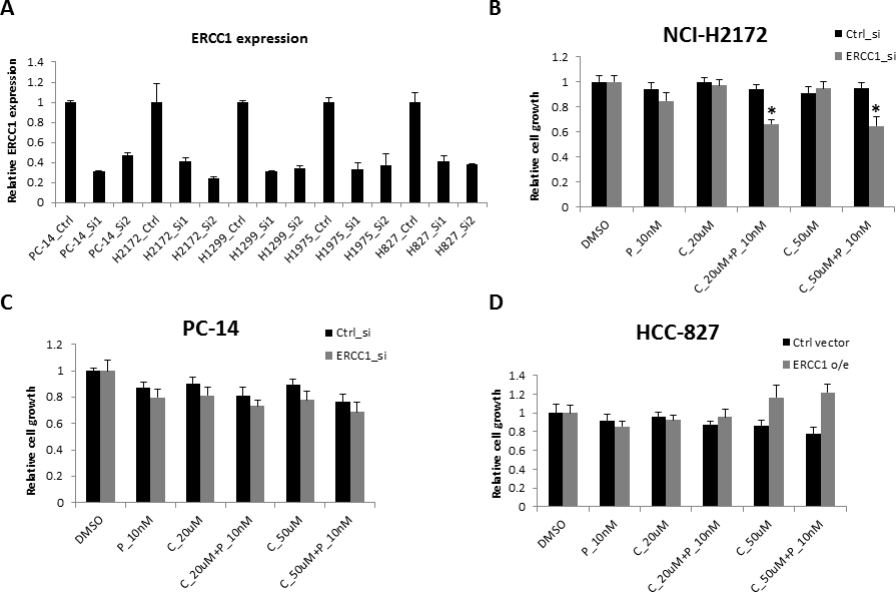
Cell sensitivity to the combination of panobinostat and cisplatin with ERCC1 knockdown **A.** NCI-H1299, NCI-H2172 and PC14 cells were transfected with two independent siRNAs against ERCC1. The KD efficiency of these two siRNAs was confirmed using RT-qPCR after a 48-hour transfection. The data are expressed as the mean ± SD. After a 24-hour transfection, all three cell lines were treated with 10 nM panobinostat combined with different doses of cisplatin for 48 hours. Cell viability was assessed using Cell-Titer Glo. The data are expressed as the mean ± SD*, *p* < 0.05, *t* test. The data plots are as follows: **B.** NCI-H2172 and **C.** PC14. **D.** HCC-827 cells were transfected with an ERCC1 over-expression construct or pcDNA3 vector control. Cell growth was assessed 3 days after transfection. The data are expressed as the mean ± SD. C represents cisplatin and P represents panobinostat. All the values are from the average of two independent experiments.

Next, we sought to determine whether cell sensitivity to cisplatin and panobinostat was dependent on ERCC1 expression levels. First, we transfected two individual siRNAs that had been reported previously [21, 22] into PC-14, H1299 and H2172 cells, which are all ERCC1^High^ cell lines. We examined ERCC1 mRNA levels using RT-qPCR after siRNA transfection. Both siRNAs knocked down ERCC1 mRNA expression to levels lower than 50% (Fig. 3A). Then, we chose siRNA-1 to examine cell growth after ERCC1 knockdown (KD) in the setting of cisplatin and panobinostat treatment. H2172 (Fig. 3B) and H1299 (Supplementary Fig. S2A) cells became more sensitive to combination treatment after ERCC1 KD. However, we did not observe this effect in PC14 cells (Fig. 3C). In contrast, the ERCC1^Low^ H827 and H23 cell lines, which were sensitive to the panobinostat and cisplatin combination therapy, became resistant to combination treatment after ERCC1 over-expression (Fig. 3D and Supplementary Fig. S2B). These results suggest that the ERCC1 expression level affects the sensitivity of most cells to cisplatin combined with panobinostat.

### Panobinostat sensitizes NSCLC cells to cisplatin by inducing apoptosis through p53

**Figure 4:**
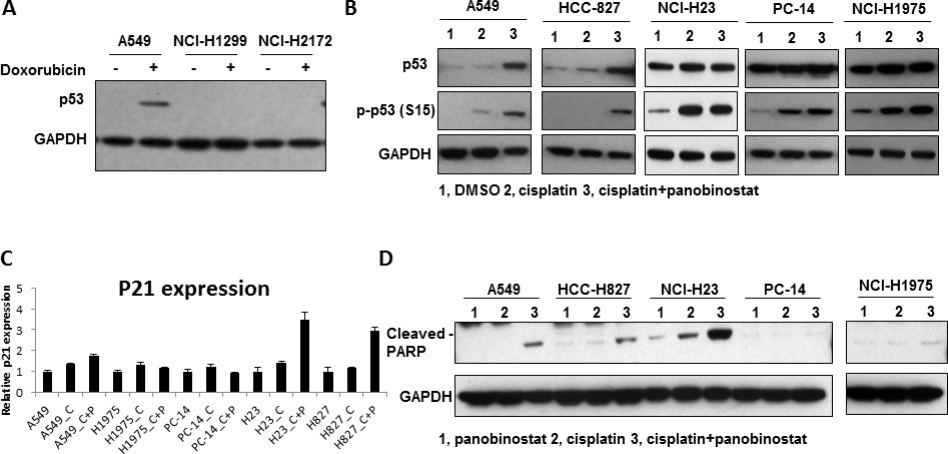
The induction of apoptosis by the combination of panobinostat and cisplatin **A.** p53 expression in H1299, H2172 and A549 cells. **B.** Panobinostat and cisplatin combination treatment increases p53 S15 phosphorylation. A549, HCC827, NCI-H23, NCI-H441, NCI-H1975 and PC14 cells were harvested for western blot analysis of p53 phosphorylation (S15). **C.** The p21 mRNA level was measured by using RT-qPCR after panobinostat and cisplatin treatment for 48 hours. C represents 50 uM cisplatin and P represents 10 nM panobinostat. **D.** Cleaved PARP levels were increased in A549, HCC827 and NCI-H23 cells. The cleaved PARP level was measured by western blot.

To further understand the mechanism of panobinostat sensitization to cisplatin, we examined the p53 pathway and apoptosis in the cell lines. H1299 and H2172, which are p53^Null^ cells, showed no p53 expression on our western blots, even after exposure to doxorubicin, which is known to be a strong p53-induction compound. We used A549 cells as a positive control to show p53 expression (Fig. 4A). Thus, we performed the p53-related apoptosis assays in the other cell lines. Phospho-p53 (S15) was found to be induced in all 5 of the cell lines analyzed (Fig. 4B). However, the induction of p21 (Fig. 4C) and cleaved poly ADP ribose polymerase (PARP) (Fig. 4D) was only observed in A549, H827 (p53^WT^/ERCC1^Low^) and H23 cells (p53^MU^/ERCC1^Low^), but not in H1975 (p53^GOF^/ERCC1^Low^) or PC14 (p53^GOF^/ERCC1^High^) cells. Flow cytometry data confirmed a significant increase in apoptosis from 3.6% to 57.5% in A549 cells after combined treatment (Supplementary Fig. S3). These results suggest that p53-related apoptosis was involved in the combination treatment, but apoptosis was not activated in H1975 and PC14 cells because both harbor GOF mutant p53.

### ERCC1 expression as well as p53 status affects cell sensitivity to cisplatin combined with panobinostat

**Figure 5:**
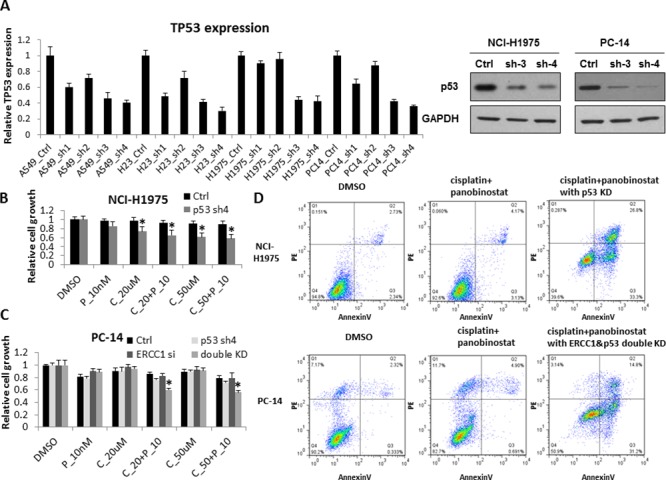
p53 contributes to increased sensitivity to cisplatin combined with panobinostat **A.** A548, HCC827, NCI-H23, NCI-H1975 and PC14 cells were transfected with four independent shRNA constructs against p53. The KD efficiency of these four shRNAs was confirmed by RT-qPCR after a 48-hour transfection. The data are expressed as the mean ± SD. The p53 KD efficiency was also confirmed by western blot. **B.** After p53 KD, NCI-H1975 cells were treated with 10nM panobinostat combined with different doses of cisplatin for 48 hours. Cell growth was measured 2 days after treatment. The data are expressed as the mean ± SD*, *p* < 0.05, *t* test. All the values are from the average of two independent experiments. **C.** PC14 cells were transfected with both ERCC1 siRNA and p53 shRNA. After a 24-hour transfection, the cells were treated with 10nM panobinostat combined with different doses of cisplatin for 48 hours. Cell growth was measured 2 days after transfection. The data are expressed as the mean ± SD*, *p* < 0.05, *t* test. All the values are from the average of two independent experiments. **D.** With the panobinostat and cisplatin combined treatment, H1975 cells with p53 knockdown and PC14 cells with both p53 and ERCC1 KD showed significantly increased levels of apoptosis by flow cytometry assay. C represents cisplatin and P represents panobinostat.

PC14 and H1975 cells have been reported to harbor GOF p53 mutations, specifically the R248Q mutation in PC14 cells and the R273H mutation in H1975 cells. To investigate whether GOF p53 mutations play a role in chemo-resistance, we knocked down p53 expression in H1975 and PC14 cells as well as in H827 cells as a wild-type p53 control. We validated the KD efficiency of the p53 shRNAs using RT-qPCR and western blot (Fig. 5A). ERCC1 expression levels did not change with p53 KD (Supplementary Fig S4A). Based on the results, p53-sh3 and p53-sh4 showed good KD efficiency. We chose p53-sh4 to examine cell proliferation in response to the combination treatment in the context of p53 KD. In A549 (ERCC1^Low^/p53^WT^) cells (Supplementary Fig. S4B) and NCI-H441 (ERCC1^Low^/p53^MU^) cells (Supplementary Fig. S4C), p53 KD did not cause any change in cell sensitivity to cisplatin combined with panobinostat. In contrast, H1975 (ERCC1^Low^/p53^GOF^) cells (Fig. 5B) showed significantly increased sensitivity to cisplatin combined with panobinostat after GOF p53 KD. Flow cytometry also showed increased apoptosis in H1975 cells after p53 KD (Fig. 5D). However, we still did not observe a significant change in the sensitivity of PC14 cells (Fig. 5C). Considering the high level of ERCC1 expression in PC14 cells, next we performed ERCC1 and p53 double KD in PC14 cells. Cell proliferation (Fig. 5C) and flow cytometry (Fig. 5D) both showed that the combination of panobinostat and cisplatin could inhibit cell growth and increase apoptosis in PC14 cells.

To further confirm the contribution of p53 to sensitivity to combined treatment, we over-expressed wild-type p53 in H2172 (ERCC1^High^/p53^Null^) cells (Supplementary Fig. S4D). The cells were then treated with cisplatin and panobinostat. Wild-type p53-transfected H2172 cells showed increased sensitivity to combined treatment. These results suggest that wild-type p53 contributes to cell sensitivity to combination cisplatin and panobinostat treatment and that GOF p53 mutations increase cell resistance to the treatment.

## DISCUSSION

Many novel drugs, such as anti-angiogenesis monoclonal antibodies (mAbs), molecular tyrosine kinase inhibitors (TKIs) and immune checkpoint inhibitors have emerged in NSCLC treatment over the last 10 years. However, platinum-based regimens are still preferred in NSCLC adjuvant treatment and are first-line therapy in advanced NSCLC. Almost all regimens eventually fail due to the development of drug resistance. Therefore, enhancing drug sensitivity, reversing drug resistance and prolonging the time to drug resistance are very important research goals.

Cisplatin is a chemotherapeutic drug that binds to and crosslinks DNA, leading to the induction of cancer cell apoptosis. The anticancer effect of cisplatin is increased by HDACis, and the underlying mechanisms have been investigated since 2003. Many mechanisms contribute to the synergistic effect of combined treatment [21, 23–26]. Among them, the chromatin structure alterations induced by HDACis, which make DNA double strands more accessible to covalent modification by cisplatin, appear to be the major mechanism responsible for sensitizing cancer cells to cisplatin. It has been reported that combination of cisplatin and panobinostat can overcome hypoxia-induced cisplatin resistance in NSCLC cells [15].

Furthermore, some studies have found that up-regulation of apoptotic proteins such as p53, Bim and caspase 3 and down-regulation of glutathione (GSH) enhance cisplatin-induced cytotoxicity in oral squamous cell carcinoma (OSCC) cells. Since vorinostat and romidepsin were approved for use in cutaneous T-cell lymphoma (CTLC) by the US FDA, several similar drugs have been evaluated in NSCLC treatment in recent years [27]. For example, entinostat and chidamide have entered phase II clinical trials, and vorinostat and panobinostat have been studied in phase III clinical trials [10, 11].

The identification of biomarkers is one of the most important aspects of studying a new therapeutic class. It is necessary to identify which patients may benefit from a particular therapy. However, to date, predictable biomarkers for selecting patients or predicting responses to HDACi treatment remain undetermined. Some studies have reported that histone 3 and 4 hyperacetylation (because an accumulation of acetylated histones is a direct downstream consequence of HDAC inhibition) and HR23B (a protein that transports ubiquitinated cargo to proteasomes) are biomarkers for sensitivity to HDACi-induced apoptosis [28, 29]. However, those biomarkers are not yet widely used, and their reliability in clinical practice remains to be confirmed. Here, we investigated the predictive role of two classical and readily assayable biomarkers, ERCC1 and p53, which are involved in the underlying mechanisms of NSCLC cell treatment with a combination of HDACis and cisplatin.

Although the association between cisplatin sensitivity and ERCC1 expression level was not as strong in our eight NSCLC cell lines as in previous reports, the present study found that ERCC1 expression had a very clear predictive role with regard to the efficacy of panobinostat and cisplatin combination therapy. Notably, we used 10 nM panobinostat, which is a concentration that is not typically employed in cancer treatment but is used as a sensitizer to cisplatin to avoid the toxicity of non-specific HDACis. Improved cytotoxicity was only observed in the four NSCLC cell lines with low ERCC1 expression, with the exception of the H1975 cell line, which also contains a GOF p53 mutation. None of the other three cell lines with high ERCC1 expression levels showed increased sensitivity to panobinostat and cisplatin combination treatment. Next, we knocked down ERCC1 in these three cell lines with high ERCC1 expression. The two p53^Null^ cell lines but not PC14 (ERCC1^High^/p53^GOF^) became sensitive to cisplatin and panobinostat combination treatment. These results indicated that ERCC1 can be used as an effective biomarker for combination therapy with HDACis and cisplatin, and the mechanism may be related to the DNA repair capability determined by the ERCC1 level. However, the data also implied that GOF p53 mutations might contribute to resistance to panobinostat and cisplatin combination treatment.

Our above results suggest that p53 mutation status is very important in the response of NSCLC cell lines to panobinostat and cisplatin combination treatment. It has also been reported that GOF mutant p53 can lead to drug resistance by inhibiting cancer cell apoptosis [30, 31]. Thus, we further investigated the association between p53 status and drug resistance. We used shRNA to knock down the expression of GOF mutant p53 in H1975 (ERCC1^Low^/p53^GOF^) and PC14 (ERCC1^High^/p53^GOF^) cell lines. After KD of GOF mutant p53, the H1975 cells became sensitive, whereas the PC14 cells remained resistant to cisplatin and panobinostat combination treatment. However, it is very interesting that double KD of ERCC1 and GOF mutant p53 in the PC14 cell line significantly increased the sensitivity to combination panobinostat and cisplatin treatment. These results demonstrated that GOF p53 mutation as well as a high ERCC1 expression level plays important roles in the resistance of NSCLC cell lines to panobinostat and cisplatin combination treatment. Therefore, based on these findings, combined screening for GOF p53 mutations and ERCC1 expression level could be more valuable than either individual biomarker in predicting the efficacy of panobinostat and cisplatin combination treatment.

HDACis not only loosen the structure of chromosomes to allow DNA-damaging drugs such as cisplatin to come into contact with the DNA strands more easily, but they also up-regulate apoptotic proteins such as p53 and p21. Our western blotting showed that the p21 and PARP levels were increased in the two p53^WT^ and the two p53^MU^ cell lines, which all showed increased sensitivity to combination treatment. However, in the two p53^GOF^ cell lines (H1975 and PC14) that were still resistant to combination treatment, no p21 or PARP was induced. Our study indicated that HDACis could significantly improve the cytotoxicity of cisplatin only in low ERCC1-expressing NSCLC cell lines without GOF p53 mutations based not only on chromatin structure alterations but also on p53-induced cell apoptosis. We confirmed that high expression levels of ERCC1 and GOF p53 mutants can contribute to the resistance of NSCLC cell lines to the above treatment, and combined screening for these two biomarkers might have great potential value in future clinical practice.

## MATERIALS AND METHODS

### Cell culture and proliferation assay

Human lung cancer cell lines A549 (ATCC Cat#: CCL-185), NCI-H23 (ATCC Cat#: CRL-5800), NCI-H1299 (ATCC Cat#: CRL-5803), NCI-H441 (ATCC Cat#: HTB-174), HCC827 (ATCC Cat#: CRL-2868), NCI-H1975 (ATCC Cat#: CRL-5908) and NCI-H2172 (ATCC Cat#: CRL-5930) were obtained from American Type Culture Collection (ATCC) and cultured as instructed. The human lung cancer PC14 cell line was kindly provided by Mr. Yu (The Shanghai Cancer Institute, China). The cancer cells were maintained in Roswell Park Memorial Institute (RPMI)1640 medium supplemented with 10% fetal bovine serum (FBS) and 1% penicillin and streptomycin (all from Invitrogen). Cells were maintained under a standard gas atmosphere of humidified air/5% CO_2_.

Cell growth was measured using CellTiter-Glo (Promega). The cells were seeded in 96-well plates in 100 μl of medium with different drug doses. Cisplatin (C2210000, Sigma Aldrich) and panobinostat (EPI009, Sigma Aldrich) were dissolved in dimethyl sulfoxide (DMSO). After the cells were cultured for 48 hours, 100 μl of CellTiter-Glo reagent was added to each well to measure cell growth according to the manufacturer's instructions.

### RNA interference

The shRNA against p53 was obtained from Sigma (Clone ID: NM_000546.4-887s21c1). The following sequence was used: CCGGCACCATCCACTACAACTA CATCTCGAGATGTAGTTGTAGTGGATGGTGTTTTTG. Two independent siRNAs were used to KD ERCC1. The respective sequences were as follows: si-Q1, 5′-CAGCATGCGAATTCTGGGCAA-3′; si-Q2, 5′-TCGGGTGGTCGCCAAATACAA-3′; Ctrl-siRNA, siGLO RISC-Free Control siRNA (Dharmacon). The siRNAs were transfected using Lipofectamine^TM^ RNAiMAX (Invitrogen), and the cells were assayed 3 days after transfection.

### RNA isolation and real-time RT-qPCR

RNA was extracted using an RNeasy Mini Kit (Qiagen). The RNA quality was confirmed by Nanodrop spectrophotometry. Reverse transcription was performed according to the manufacturer's instructions using an RT Kit (Invitrogen). Real-time PCR analysis was performed on an ABI Prism 7900 Sequence Detection System using the SYBR Green PCR Master Mix (Applied Biosystems, Foster City, USA). The relative expression of each gene was normalized to glyceraldehyde 3-phosphate dehydrogenase (GAPDH). The primers used for the quantitative RT-PCR are shown in Supplementary Table S2.

### Western blot analysis

Western blot was performed as described previously. Samples were collected directly in 1X NuPAGE LDS sample buffer with 1X sample-reducing buffer (Invitrogen) and denatured at 95°C for 5 min followed by a centrifugation at 13200 rpm for 5 min. The supernatant was electrophoresed on a 4–12% Tris-HCl gel and transferred to nitrocellulose membranes (Invitrogen). After blocking with Superblock T20 blocking buffer (Thermo Scientific), the membranes were incubated with a primary antibody overnight at 4°C and then with a secondary antibody conjugated with alkaline phosphatase for 1 hour each at room temperature; the signal was detected using a chemiluminescence method. The following primary antibodies were used: anti-p53 (Cell Signaling, 1:1000); anti-phospho-p53 (S15; Cell Signaling, 1:1000); anti-GAPDH (Santa Cruz, 1:10000); anti-ERCC1 (Cell Signaling, 1:1000); and anti-PARP (Cell Signaling, 1:1000).

### Flow cytometry

Cells were plated in 6-cm dishes and exposed to compounds with or without p53 and ERCC1 KD. After 2 days of drug exposure, the cells were collected and stained using propidium iodide solution and Annexin V (BD). The cells were quantified and analyzed by flow cytometry on a fluorescence-activated cell scan cytometer.

## SUPPLEMENTAY FIGURES AND TABLES



## References

[1] Govindan R, Page N, Morgensztern D, Read W, Tierney R, Vlahiotis A, Spitznagel EL, Piccirillo J (2006). Changing epidemiology of small-cell lung cancer in the United States over the last 30 years: analysis of the surveillance, epidemiologic, and end results database. Journal of clinical oncology: official journal of the American Society of Clinical Oncology.

[2] Black WC (2007). Computed tomography screening for lung cancer: review of screening principles and update on current status. Cancer.

[3] Kristeleit R, Stimson L, Workman P, Aherne W (2004). Histone modification enzymes: novel targets for cancer drugs. Expert opinion on emerging drugs.

[4] Kim MS, Blake M, Baek JH, Kohlhagen G, Pommier Y, Carrier F (2003). Inhibition of histone deacetylase increases cytotoxicity to anticancer drugs targeting DNA. Cancer research.

[5] Namdar M, Perez G, Ngo L, Marks PA (2010). Selective inhibition of histone deacetylase 6 (HDAC6) induces DNA damage and sensitizes transformed cells to anticancer agents. Proceedings of the National Academy of Sciences of the United States of America.

[6] Grant S, Easley C, Kirkpatrick P (2007). Vorinostat. Nature reviews Drug discovery.

[7] VanderMolen KM, McCulloch W, Pearce CJ, Oberlies NH (2011). Romidepsin (Istodax, NSC 630176, FR901228, FK228, depsipeptide): a natural product recently approved for cutaneous T-cell lymphoma. The Journal of antibiotics.

[8] Robert T, Vanoli F, Chiolo I, Shubassi G, Bernstein KA, Rothstein R, Botrugno OA, Parazzoli D, Oldani A, Minucci S, Foiani M (2011). HDACs link the DNA damage response, processing of double-strand breaks and autophagy. Nature.

[9] Stiborova M, Eckschlager T, Poljakova J, Hrabeta J, Adam V, Kizek R, Frei E (2012). The synergistic effects of DNA-targeted chemotherapeutics and histone deacetylase inhibitors as therapeutic strategies for cancer treatment. Current medicinal chemistry.

[10] Drummond DC, Noble CO, Kirpotin DB, Guo Z, Scott GK, Benz CC (2005). Clinical development of histone deacetylase inhibitors as anticancer agents. Annual review of pharmacology and toxicology.

[11] Marks PA (2010). The clinical development of histone deacetylase inhibitors as targeted anticancer drugs. Expert opinion on investigational drugs.

[12] Piekarz RL, Sackett DL, Bates SE (2007). Histone deacetylase inhibitors and demethylating agents: clinical development of histone deacetylase inhibitors for cancer therapy. Cancer journal.

[13] Gultekin KE, Yurdakonar MK, Yaman E, Yuce US, Yilmaz A, Alp E, Celik A, Demiroz SM, Onen HI (2013). Effects of cisplatin and panobinostat on human mesothelial (Met-5A) and malignant pleural mesothelioma (MSTO-211H) cells. Genetics and molecular research: GMR.

[14] Wang G, Edwards H, Caldwell JT, Buck SA, Qing WY, Taub JW, Ge Y, Wang Z (2013). Panobinostat synergistically enhances the cytotoxic effects of cisplatin, doxorubicin or etoposide on high-risk neuroblastoma cells. PloS one.

[15] Fischer C, Leithner K, Wohlkoenig C, Quehenberger F, Bertsch A, Olschewski A, Olschewski H, Hrzenjak A (2015). Panobinostat reduces hypoxia-induced cisplatin resistance of non-small cell lung carcinoma cells via HIF-1alpha destabilization. Molecular cancer.

[16] Gueugnon F, Cartron PF, Charrier C, Bertrand P, Fonteneau JF, Gregoire M, Blanquart C (2014). New histone deacetylase inhibitors improve cisplatin antitumor properties against thoracic cancer cells. Oncotarget.

[17] Ha K, Fiskus W, Choi DS, Bhaskara S, Cerchietti L, Devaraj SG, Shah B, Sharma S, Chang JC, Melnick AM, Hiebert S, Bhalla KN (2014). Histone deacetylase inhibitor treatment induces ‘BRCAness’ and synergistic lethality with PARP inhibitor and cisplatin against human triple negative breast cancer cells. Oncotarget.

[18] Chen RS, Ko JC, Chiu HC, Wo TY, Huang YJ, Tseng SC, Chen HJ, Huang YC, Jian YJ, Lee WT, Lin YW (2013). Pemetrexed downregulates ERCC1 expression and enhances cytotoxicity effected by resveratrol in human nonsmall cell lung cancer cells. Naunyn-Schmiedeberg's archives of pharmacology.

[19] Li R, Sutphin PD, Schwartz D, Matas D, Almog N, Wolkowicz R, Goldfinger N, Pei H, Prokocimer M, Rotter V (1998). Mutant p53 protein expression interferes with p53-independent apoptotic pathways. Oncogene.

[20] Lai SL, Perng RP, Hwang J (2000). p53 gene status modulates the chemosensitivity of non-small cell lung cancer cells. Journal of biomedical science.

[21] Cheng H, Zhang Z, Borczuk A, Powell CA, Balajee AS, Lieberman HB, Halmos B (2013). PARP inhibition selectively increases sensitivity to cisplatin in ERCC1-low non-small cell lung cancer cells. Carcinogenesis.

[22] Chang IY, Kim MH, Kim HB, Lee DY, Kim SH, Kim HY, You HJ (2005). Small interfering RNA-induced suppression of ERCC1 enhances sensitivity of human cancer cells to cisplatin. Biochemical and biophysical research communications.

[23] Marks PA, Xu WS (2009). Histone deacetylase inhibitors: Potential in cancer therapy. Journal of cellular biochemistry.

[24] Haberland M, Montgomery RL, Olson EN (2009). The many roles of histone deacetylases in development and physiology: implications for disease and therapy. Nature reviews Genetics.

[25] Wu J, Hu CP, Gu QH, Li YP, Song M (2010). Trichostatin A sensitizes cisplatin-resistant A549 cells to apoptosis by up-regulating death-associated protein kinase. Acta pharmacologica Sinica.

[26] Luchenko VL, Salcido CD, Zhang Y, Agama K, Komlodi-Pasztor E, Murphy RF, Giaccone G, Pommier Y, Bates SE, Varticovski L (2011). Schedule-dependent synergy of histone deacetylase inhibitors with DNA damaging agents in small cell lung cancer. Cell cycle.

[27] Wang H, Dymock BW (2009). New patented histone deacetylase inhibitors. Expert opinion on therapeutic patents.

[28] La Thangue NB, Kerr DJ (2011). Predictive biomarkers: a paradigm shift towards personalized cancer medicine. Nature reviews Clinical oncology.

[29] Giannini G, Cabri W, Fattorusso C, Rodriquez M (2012). Histone deacetylase inhibitors in the treatment of cancer: overview and perspectives. Future medicinal chemistry.

[30] Oren M, Rotter V (2010). Mutant p53 gain-of-function in cancer. Cold Spring Harbor perspectives in biology.

[31] Solomon H, Madar S, Rotter V (2011). Mutant p53 gain of function is interwoven into the hallmarks of cancer. The Journal of pathology.

