# Supplementary material for: The predictive value of ERCC1 and p53 for the effect of panobinostat and cisplatin combination treatment in NSCLC
Source: Oncotarget. 2015 Apr 29;6(22):18997–9005. doi: 10.18632/oncotarget.3620 (PMC4662470; doi:10.18632/oncotarget.3620)
Supplement: Supplementary file 1 [file oncotarget-06-18997-s001.pdf]

SUPPLEMENTARY FIGURES AND TABLES

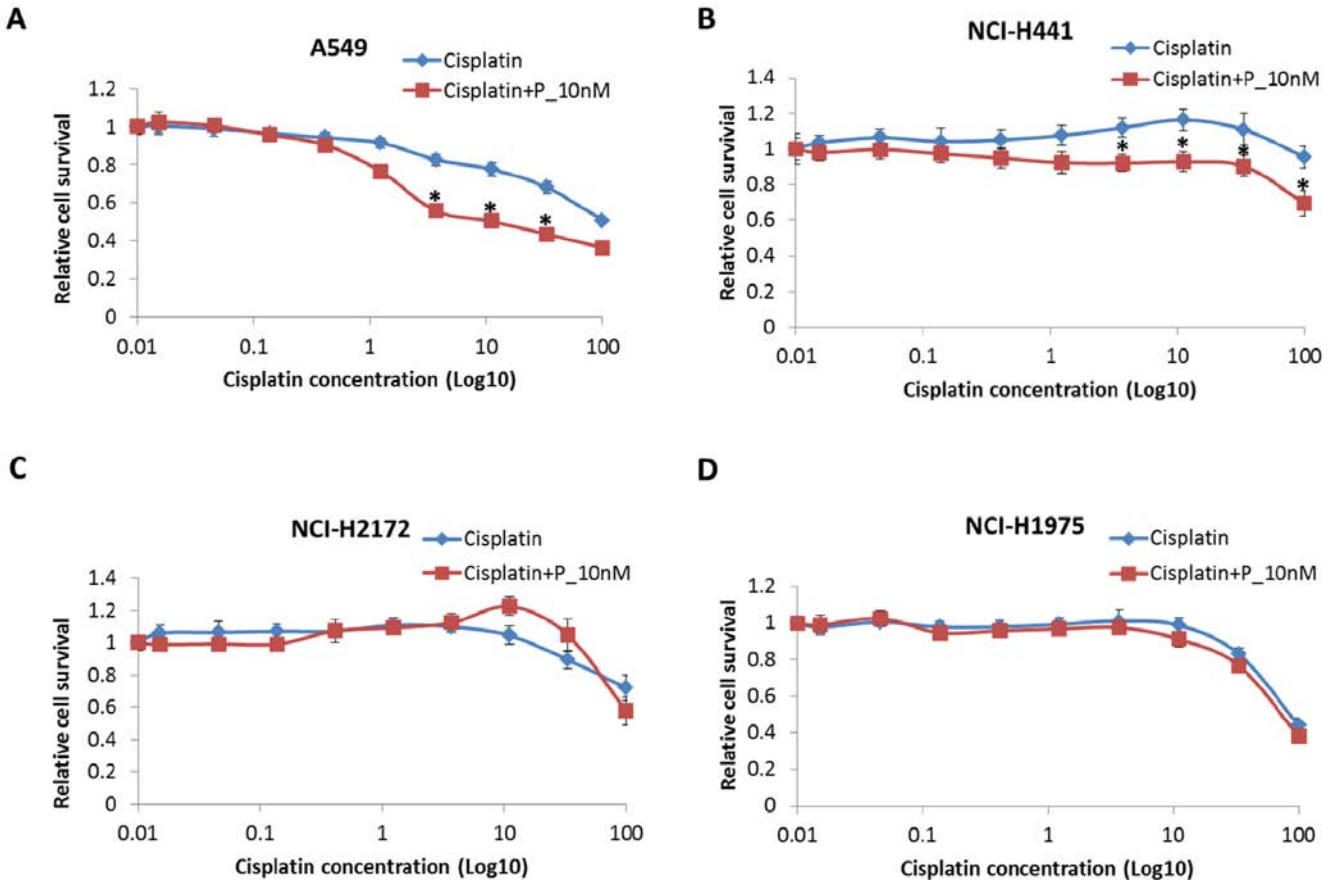

Supplementary Figure S1: Cell sensitivity to cisplatin and panobinostat combined with cisplatin. Cells were treated with 10 nM panobinostat combined with different doses of cisplatin for 48 hours. Cell viability was assessed using Cell-Titer Glo. The data are expressed as the mean ± SD. P represents panobinostat. The data plots are as follows: **A.** A549 cells; **B.** NCI-H441 cells; **C.** NCI-H2172 cells; and **D.** NCI-H1975 cells. P represents panobinostat. The data are expressed as the mean ± SD*, $p < 0.05$, *t* test. The values are from the average of three independent experiments.

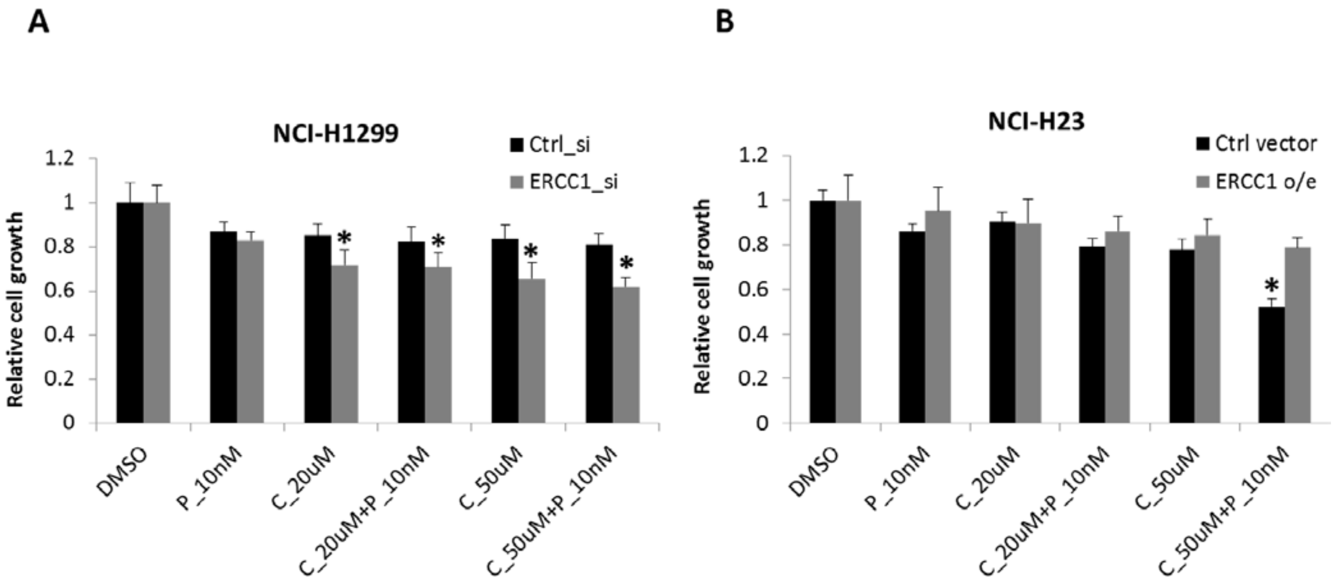

Supplementary Figure S2: Sensitivity to the combination of panobinostat and cisplatin in ERCC1 knockdown or over-expressing cells. A. NCI-H1299 cells were transfected with ERCC1 siRNA for 24 hours followed by treatment with 10 nM panobinostat combined with different doses of cisplatin for 48 hours. Cell viability was assessed using Cell-Titer Glo. The data are expressed as the mean ± SD*, $p < 0.05$, *t* test. C represents cisplatin, and P represents panobinostat. **B.** NCI-H23 cells were transfected with ERCC1 over-expression construct or pcDNA3 vector control. Cell growth was assessed 3 days after transfection. The data are expressed as the mean ± SD*, $p < 0.05$, *t* test. C represents cisplatin, and P represents panobinostat. All the values are from the average of two independent experiments.

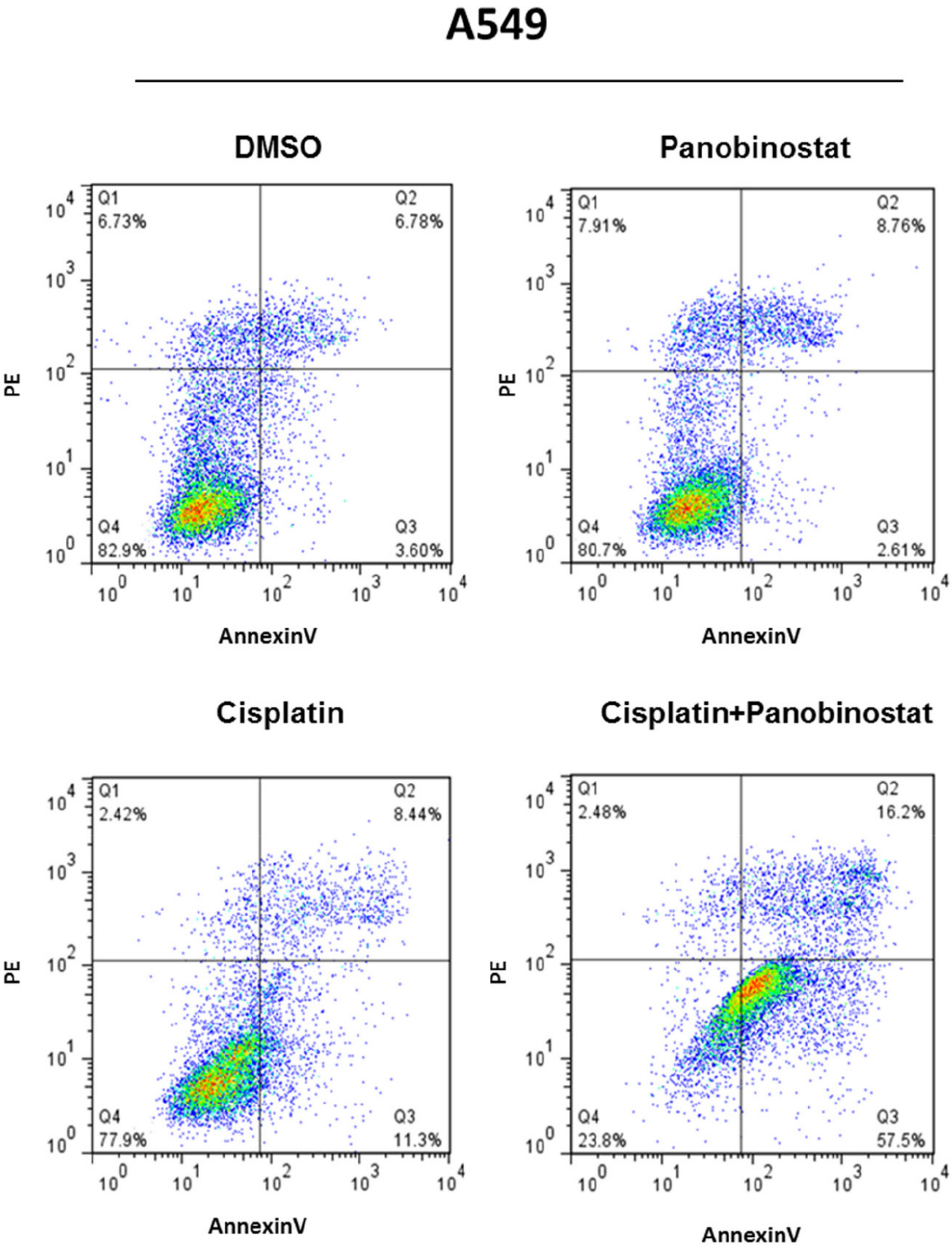

Supplementary Figure S3: The induction of apoptosis by the combination of panobinostat and cisplatin. Cell apoptosis was significantly increased in A549 cells after panobinostat and cisplatin combination treatment as determined via flow cytometry.

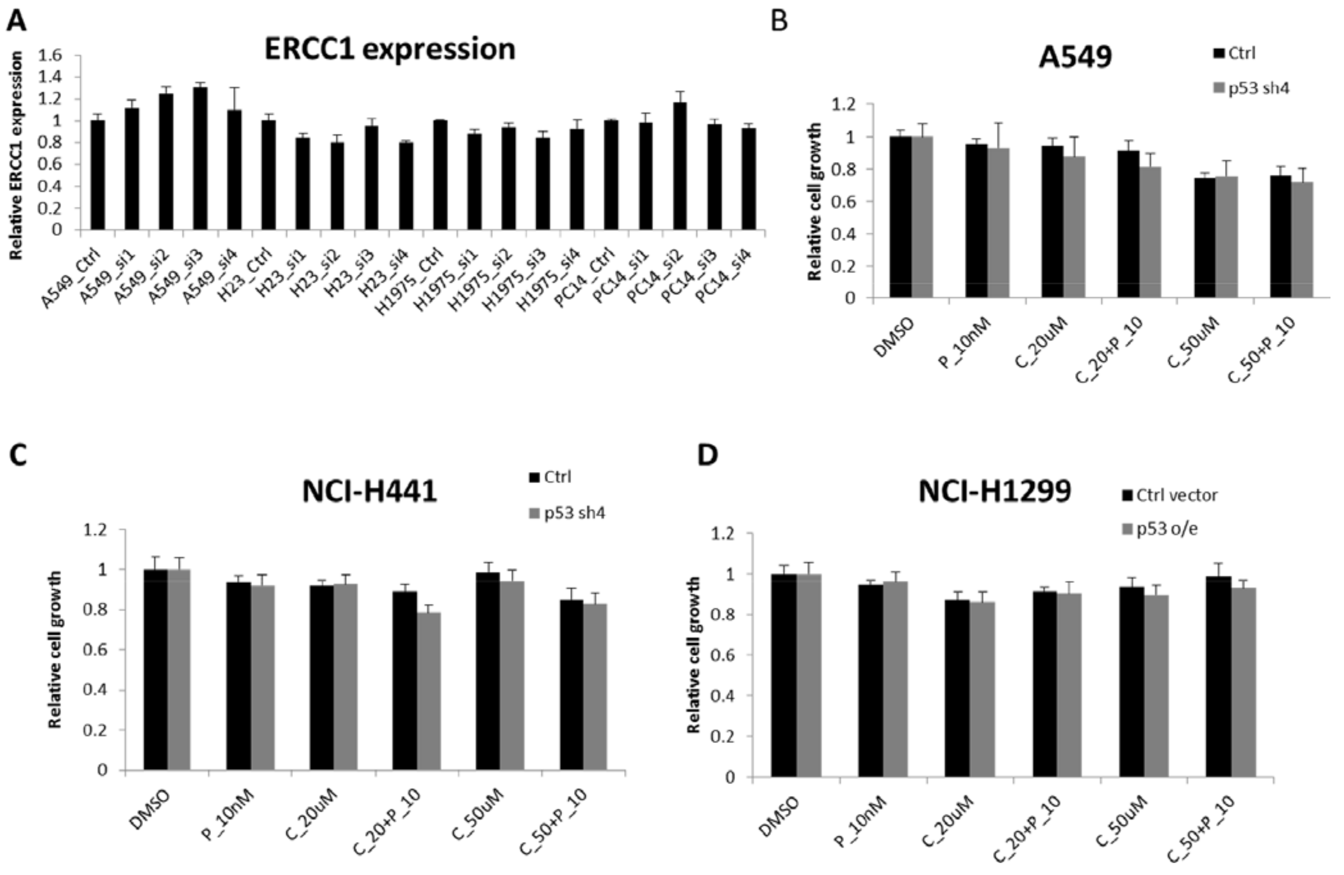

Supplementary Figure S4: P53 contributes to the increased sensitivity to cisplatin combined with panobinostat. A. The ERCC1 expression level after p53 knockdown was confirmed using RT-qPCR after a 48-hour transfection. The data are expressed as the mean ± SD. **B.** A549 and **C.** NCI-H441 cells were transfected with p53 shRNA4 for 24 hours followed by treatment with 10 nM panobinostat combined with different doses of cisplatin for 48 hours. Cell growth was measured 2 days after treatment. The data are expressed as the mean ± SD. **D.** H2172 cells were transfected with wild-type p53 construct for 24 hours followed by treatment with 10 nM panobinostat combined with different doses of cisplatin for 48 hours. Cell growth was measured 2 days after treatment. The data are expressed as the mean ± SD*, $p < 0.05$. C represents cisplatin and P represents panobinostat. C_50 represents 50 uM cisplatin and P_10 represents 10 nM panobinostat. All the values are from the average of two independent experiments.

Supplementary Table S1. ERCC1 expression level and p53 status in 8 NSCLC cell lines

<table>
<tr><th>Cell line</th><th>ATCC Cat#</th><th>ERCC1 expression</th><th>p53 status</th><th>Abbreviated formof gene status</th></tr>
<tr><td>A549</td><td>CCL-185</td><td>Low</td><td>WT</td><td rowspan="2">$ERCC1^{Low}/P53^{WT}$</td></tr>
<tr><td>HCC827</td><td>CRL-2868</td><td>Low</td><td>WT</td></tr>
<tr><td>PC-14</td><td>/</td><td>High</td><td>R248Q (GOF)</td><td>$ERCC1^{High}/P53^{GOF}$</td></tr>
<tr><td>NCI-H1975</td><td>CRL-5908</td><td>Low</td><td>R273H (GOF)</td><td>$ERCC1^{Low}/P53^{GOF}$</td></tr>
<tr><td>NCI-H23</td><td>CRL-5800</td><td>Low</td><td>M246I</td><td rowspan="2">$ERCC1^{Low}/P53^{MU}$</td></tr>
<tr><td>NCI-H441</td><td>HTB-174</td><td>Low</td><td>R158L</td></tr>
<tr><td>NCI-H1229</td><td>CRL-5803</td><td>High</td><td>Null</td><td rowspan="2">$ERCC1^{High}/P53^{Null}$</td></tr>
<tr><td>NCI-H2172</td><td>CRL-5930</td><td>High</td><td>Null</td></tr>
</table>

Supplementary Table S2. Sequence of the primers used in the study

RT qPCR primers	sequences (5′—3′)
GAPDH	TGACAACAGCCTCAAGATCA
	CTGTGGTCATGAGTCCTTCC
ERCC1	TTGTCCAGGTGGATGTGAAA
	GCTGGTTTCTGCTCATAGGC
P53	GTTCCGAGAGCTGAATGAGG
	TCTGAGTCAGGCCCTTCTGT
P21	ATGCCTTCCGATCAGTACGAG
	CGACCACGTAGATAGTGCTGT
